# Regulation of Stemness by NR1D2 in Colorectal Cancer

**DOI:** 10.3390/biomedicines13061500

**Published:** 2025-06-18

**Authors:** Sandra Alonso-García, Paula Sánchez-Uceta, Sara Moreno-SanJuan, Jorge Casado, Jose D. Puentes-Pardo, Huda Khaldy, David Lopez-Pérez, María Sol Zurita-Saavedra, Cristina González-Puga, Angel Carazo, Josefa León

**Affiliations:** 1 Unidad de Gestión Clínica de Cirugía, Hospital Clínico Universitario San Cecilio, 18012 Granada, Spain; 2 Instituto de Investigación Biosanitaria de Granada(ibs.GRANADA), 18012 Granada, Spain; 3 Servicio de Microscopía y Citometría, Instituto de Investigación Biosanitaria de Granada(ibs.GRANADA), 18012 Granada, Spain; 4 Servicio de Biología Fundamental, Centro de Instrumentación Científica, Universidad de Granada, 18071 Granada, Spain; 5 Laboratory of Cell Biology, National Cancer Institute, National Institutes of Health, Bethesda, MD 20892, USA; 6 Unidad de Gestión Clínica de Microbiología, Hospital Universitario San Cecilio de Granada, 18016 Granada, Spain; 7 Unidad de Gestión Clínica de Aparato Digestivo, Hospital Clínico Universitario San Cecilio, 18016 Granada, Spain

**Keywords:** colorectal cancer, cancer stem cells, circadian clock, NR1D2, p53

## Abstract

**Background**: Nuclear Receptor Subfamily 1 Group D Member 2 (NR1D2), a transcription factor that regulates the circadian clock, has been described as an oncogene in colorectal cancer (CRC). In several types of cancer, NR1D2 regulates cancer progression and relapse through cancer stem cells (CSCs), although this aspect has not been studied in CRC. On the other hand, p53 is a tumour suppressor gene that appears mutated in approximately a 50% CRCs. Interestingly, p53 is considered to be a crucial nexus between circadian clock deregulation and cancer. In addition, p53 regulates CSC phenotypes. **Methods**: We developed an in vitro model in which NR1D2 was silenced in three isogenic cell lines with different p53 status. In addition, we analysed the expression of NR1D2 in a cohort of patients and determined its relationship with the characteristics of patients and tumours. **Results**: In the in vitro model, NRID2 silencing reduces cell growth and decreases stemness, although only in cells harbouring a wild type p53. In contrast, in cells lacking a functional p53 or harbouring a mutated one, NR1D2 knockout increases cell growth and stemness. In patients, NR1D2 expression correlates with poorly differentiated tumours and high expression of CSCs markers, although only in tumours with a wild type p53, corroborating the results obtained in the in vitro model. **Conclusions**: Although more research is needed to analyse the mechanism by which NR1D2 regulates stemness in a p53-dependent manner, our results highlight the possibility of using NR1D2 antagonists for treating this type of patient and to develop personalised medicine.

## 1. Introduction

Colorectal cancer is the third most common cancer worldwide in terms of incidence and the second leading cause of cancer-related deaths globally [1]. Approximately a 35–55% of patients will present metastasis, the main cause of morbidity and mortality [2]. Locoregional recurrence in CRC is diagnosed in approximately 4–12% of patients who have undergone curative resection of stage I–III primary colon cancer [3]. In addition, therapy resistance is another major problem in these patients, decreasing tumour response and quality of life [4].

Cancer stem cells (CSCs), or cancer initiating cells (CICs), are a subpopulation of cells within a tumour that can self-renew and differentiate into various cell types that include the tumour. CSCs reside within a specialised microenvironment, also known as the tumour niche, that is composed of several cellular and non-cellular components that dynamically interact with CSCs, modifying their functions, and vice versa. This microenvironment provides biochemical and mechanical signals that support CSC maintenance, proliferation, and plasticity [5]. Several pathways such us Wnt/β-catenin, p53, Notch, PI3K/mTOR, Hedgehog, and TGF-β are implicated in of stem-like features and self-renewal in colorectal CSCs [6]. In particular, p53 is a major regulator of cancer stemness. P53 inactivating mutations in tumours lead to increased expression of CSC markers and sphere forming ability. In addition, certain p53 missense mutants further promote these phenotypes aggravating the malignant condition [7]. CSCs are believed to be pivotal in tumour initiation, progression, metastasis development, relapse and treatment resistance [4]. Therefore, it is crucial to investigate new regulatory mechanisms of CSCs and their potential as treatment targets.

The circadian clock is the biological timing mechanism that governs endocrine, metabolic, and immune functions to regulate physiological homeostasis [8]. In mammals, the circadian system is hierarchically organised, with the master clock located in the suprachiasmatic nucleus (SCN) of the hypothalamus. The SCN receives direct retinal innervation via the retino-hypothalamic tract (RHT) to synchronise with day–night cycles. The SCN clock induces behavioural, autonomic and neuroendocrine signals to coordinate the peripheral cellular clocks across the body, including the colon, which in turn direct local programmes of circadian gene expression that regulate physiological rhythms critical to health [9]. At the molecular level, circadian rhythms are generated through transcription/translation feed-back loops (TTFL). In the main loop, the transcription factors CLOCK and BMAL1 dimerise (CLOCK:BMAL1) and induce the expression of Period 1–3 (PER1-3) and Cryptochrome 1–2 (CRY1-2) proteins, which repress their own expression by inhibiting the transcriptional activity of CLOCK:BMAL1. A new cycle of transcription through CLOCK:BMAL1 can begin when the levels of PER and CRY proteins decrease sufficiently. In an additional loop, CLOCK:BMAL1 also induces the expression of the nuclear receptors NR1D1-2, and the retinoid-related orphan receptors α, β, and γ (RORα/β/γ). These are, respectively, transcriptional repressors and activators of BMAL1, ensuring its rhythmic expression. In addition to the core regulatory loops, other levels of epigenetic, posttranscriptional, and posttranslational regulation contribute to the molecular clockwork, finally controlling the expression of clock-controlled genes (CCGs), which mediate circadian output [10].

The dysregulation of circadian rhythms has been associated with the development of cancer and resistance to treatments [11]. Mechanistically, circadian clock components modulate cell cycle checkpoints and the tumour microenvironment (TME), and they interact with oncogenes (MYC and RAS) and the tumour suppressor gene p53 [12]. Because of this, initially, the components of the central circadian clock were described as tumour suppressor genes. However, it is now well known that these proteins can act as both oncogenes and tumour suppressor genes, depending on the type of tumour and the hallmark that is studied within the same tumour type [12]. In CRC, among the core circadian components, BMAL1, ROR, and PER members have been described as tumour suppressors, while CLOCK, NR1D, and CRY members have been described as tumour promoters [13].

Regarding NR1D1-2 proteins, we previously reported that NR1D2 is an independent prognostic factor for local recurrence in CRC [14]. However, the molecular mechanism is still unknown. In a cancer model of Drosophila, NR1D2 facilitates glioblastoma growth by maintaining the subpopulation of CSCs through the Hippo and Notch pathways [15]. In fact, the circadian clock regulates differentiation in normal stem cells and CSCs [11]. Further, the dissemination of CSCs could be regulated via the circadian clock [16]. Although signals from the TME appear to be responsible for the circadian regulation of CSCs [17], additional lines of evidence have demonstrated that the CSC population has an intact molecular clock that exhibits circadian rhythms [11].

Considering the above, the main objective of this study was to study the potential involvement of NR1D2 in regulating CSCs in CRC, as well as the influence of p53 status on this effect, using an in vitro model. Finally, we will analyse the expression of NR1D2 in a cohort of patients to determine its role in CRC progression.

## 2. Materials and Methods

### 2.1. Patients

In this study, we included 196 patients who underwent surgery for primary sporadic CRC, which correspond to a subcohort of a previous study in which 201 patients were enrolled (project code: PI-067/2013; date of approval: 24 January 2014) [18].

The inclusion criteria comprising people aged 18 years or older, diagnosed with CRC for the first time, and not due to hereditary disease, not treated with neoadjuvant therapy and not previously diagnosed with or treated for any other type of cancer. All patients gave written informed consent for the use of samples in biomedical research. tumour and adjacent non-tumour samples were collected from each of them, which were immediately dissected, included in Tissu-Teck1 (Optimal Cutting Temperature Compound, Sakura Finetek Europe B.V., Zoeterwoude) and stored for later use by the Andalusian Tumor Biobanks Network (RBTA).

### 2.2. Analysis of p53 Mutations in CRC Samples

First, DNA was extracted from tissues using QIAamp DNA Mini Kit (Qiagen, Hildem, Germany) and quantified in a NanoDrop ND-1000 (Implen GmbH, Munich, Germany). Its integrity was evaluated via electrophoresis in an agarose gel. Then, TP53 mutations in exons 2-10 of the tissues were obtained by PCR using specific primers (Table S1) [19]. PCR products were cleaned (Wizard SV gel and a PCR clean-up system; Promega, Madison, WI, USA) and sequenced (3130 XL tool; Apllied Biosystems, Foster City, CA, USA). Finally, the results were analysed with the Chromas Lite 2.1.1 (St South Brisbane, QLD, Australia) software [17].

Once obtained (Table S2), mutations were described and interpreted according to the Human Genome Variation Society (www.hgvs.org; accessed on 2 June 2025). The functional activity of mutants was obtained from The TP53 website (https://p53.fr/tp53-database; accessed on 3 June 2025), last updated January 2025 (R25, https://tp53.cancer.gov; accessed on 3 June 2025): (1) No activity: median ≤20; (2) Partial activity: median >20 and ≤75; (3) Fully active: median >75 and ≤140; (4) Hyper active: median >140; (5) No data: this mutant has not been tested. Mutants with a median transcriptional activity >75% were classified as wild-type for the calculation, while mutants with median transcriptional activity <75% were considered partially functionals and classified as mutant for the calculation [20]. In addition, any synonymous mutants affecting transcription, splicing or translation was considered and calculated as mutant for the calculation [21].

### 2.3. RNA Extraction and qPCR

To obtain total RNA from tissues or cultured cells, they were homogeneised in TRIzol reagent and extracted following the manufacturer’s recommendations (Invitrogen, Life Technologies, Carlsbad, CA, USA). The concentration was determined by UV spectrophotometry, and its integrity was obtained by agarose gel electrophoresis. The qScript™ cDNA Synthesis kit (Quanta Biosciences, Gaithersburg, MD, USA) was used to synthesise the first-strand cDNA. This was amplified using the PerfeCTa SYBR Green SuperMix Kit (Quantabio, Beverly, MA, USA). Sequence-specific primers are shown in Table S3.

### 2.4. Cell Culture and Reagents

The cell lines HCT-116 (p53 wild-type), HCT-116 p53 null (p53 silenced) and HCT-116 p53 mutated (knock-in of mutant at R248W p53 and knockout of wild-type allele) (Horizon Discovery, Cambridge, UK) were cultured in RPMI 1640 supplemented with 2 mM L-glutamine, 10% FBS, and a 1% antibiotic-antimycotic cocktail containing penicillin (100 U/mL), streptomycin (100 µg/mL), and amphotericin B (250 ng/mL) (Gibco, Carlsbad, CA, USA) at 37 °C with 5% CO_2_.

### 2.5. Transfection Protocol

Cells were plated at a density of 5 × 10^4^ cells/mL in a 6-well plate, left to attach overnight and transfected with siRNA-NR1D2 (siNR1D2) (Santa Cruz Biotechnology Inc., Dallas, TX, USA) (50 ng/mL) and lipofectamine 2000 transfection reagent (Thermo Fisher Scientific, Waltham, MA, USA) for 7 h in 1 mL transfection medium without FBS. Then, 1 mL 2X FBS-completed RPMI medium was added, and cells were maintained under these conditions overnight. Next day, the medium was changed with 1X FBS-completed RPMI, and cells were maintained under these conditions for 72 h. NR1D2 silencing was tested by immunoblotting and qPCR. Scrambled siRNA was used as a negative control.

### 2.6. Immunoblotting

Cells were washed in ice-cold PBS and incubated in RIPA lysis buffer containing protease inhibitors. Thirty-five micrograms of proteins were loaded in 10% SDS polyacrylamide gels and transferred to activated PVDF membranes. The following antibodies were used: NR1D2 (Santa Cruz Biotechnology Inc., Dallas, TX, USA), and β-actin (Santa Cruz Biotechnology Inc., Dallas, TX, USA). Secondary antibodies were visualised by enhanced chemiluminescence using HRP-conjugated secondary antibodies (Santa Cruz Biotechnology, Inc., Dallas, TX, USA). Immunoreactivity was quantified using Quantity One 4.6.8 (Bio-Rad Laboratories, Inc.) software.

### 2.7. Cell Cycle Analysis

The cells were harvested by trypsinisation and collected by centrifugation for 5 min at 200 g. After washing twice with PBS, the cells were fixed in ice-cold 70% ethanol and stained with 500 mL of PI/RNase staining solution (Immunostep S.L., Salamanca, Spain) for 30 min at room temperature in darkness. The DNA content was analysed within 1 h in a BD FACS Aria IIIu Flow Cytometer (Becton Dickinson, BD Bioscience, UK). Experiments were performed at least three times and two samples per group were analysed in each case.

### 2.8. Apoptosis Assay

This assay was performed using the IP-Annexin V kit (BD Biosciences, NJ, USA). Briefly, cells were collected, washed twice with ice-cold PBS, resuspended in 1X Binding Buffer and counted. Next, 5 µL of FITC Annexin V and 5 µL of PI were added to 10^5^ cells and incubated for 15 min at room temperature in the dark. Then, 400 µL of 1X Binding Buffer was added to each tube. Cells were analysed by flow cytometry within 1 h using the BD FACS Aria IIIu Flow Cytometer (BD Biosciences, Franklin Lakes, NJ, USA).

### 2.9. Aldefluor Assay and Cell Surface Markers Analysis

ALDH1 activity was detected using the Aldefluor assay (Stem Cell Technologies, Vancouver, BC, Canada) kit. Briefly, cells were collected, suspended in aldelfuor assay buffer containing ALDH1 substrate (BAAA, 1 μmol/L per 1 × 10^6^ cells) and incubated during for 45 min at 37 °C in darkness, with or without diethylamino benzaldehyde (DEAB). Finally, cells were analysed using a BD FACSAria III flow cytometer (BD Biosciences, Franklin Lakes, NJ, USA). Cell surface markers were obtained in percentage of marked cells by flow cytometry using a BD FACSAria III (BD Biosciences, Franklin Lakes, NJ, USA). The antibodies used were human anti-CD44-PE, anti-CD326-FITC, and anti-CD133-APC (Biolegend, San Diego, CA, USA).

### 2.10. MTT Assay

Cells were seeded in 6-well plates at a density of 5 × 10^5^ cells per well. Then, cells were transfected with siNR1D2 for 7 h in 1 mL of transfection medium without FBS. Then, 1 mL 2X FBS completed RPMI medium was added, and cells were maintained under these conditions overnight. The next day, the cells were collected and seeded in 96-well plates at a density of 4 × 10^3^ cells per well and allowed to grow under these conditions for 72 h. After this time, 0.5 mg/mL of MTT was added to each well. After 4 h, 100 μL of lysis buffer (20% SDS in 50% N, N-dimethylformamide at pH 4.7) was added. Next day, blue formazan was measured in a microplate reader (TRIAD series, Dynex Technologies Multimode Reader) at 570 nm.

### 2.11. Clonogenic Assay

Cells were seeded in 6-well plates at a density of 10^5^ cells per well. Then, cells were transfected with siNR1D2 for 7 h in 1 mL transfection medium without FBS. Then, 1 mL of 2X FBS completed RPMI medium was added, and cells were maintained under these conditions overnight. Next day, cells were collected and seeded in 6-well plates at a concentration of 1000 cells/well and left to grow under these conditions for 14 days. Then, colonies were fixed and stained with 0.5% oxalate crystal violet solution in 50% methanol for 5 min. Finally, colonies were counted manually, considering only those greater than 50 cells.

### 2.12. Spheres Formation Assay

Cells were seeded in 6-well plates at a density of 10^5^ cells per well. Then, cells were transfected with siNR1D2 for 7 h in 1 mL transfection medium without FBS. Then, 1 mL of 2X FBS completed RPMI medium was added, and cells were maintained under these conditions overnight. Then, 1.500 cells were cultured in specific conditions for sphere formation (DMEM:F12, 1% penicillin/streptomycin, B27, 10 μg/mL ITS, 1 μg/mL hydrocortisone, 4 ng/mL heparin, 10 ng/mL EGF, 20 ng/mL FGF) in 96-well plates, previously coated with poly-2-hydroxyethyl methacrylate (Merk, Darmstadt, Germany). After 3 days, onlyspheres greater than 50 μM in diameter were counted.

### 2.13. Statistical Analysis

Before to perform statistical analysis, mRNA levels of genes in tumour were normalised to normal mucosa. Continuous and categorical variables were expressed using the median and interquartile range (IQR) and numbers and percentages, respectively. To establish high and low mRNA expression cut-off, the median value was used. The association of gene expression and clinicopathological characteristics of patients was assessed with the non-parametric Kruskal–Wallis and Mann–Whitney U tests. Correlation analysis was performed using the Pearson’s test after normalisation of the variables using natural logarithms. Comparation of NR1D2 levels and CSCs markers was carried out using Fisher’s exact test. Significant differences between groups were considered when *p* < 0.05. All analyses were executed with the SPSS software version 15.0 (IBM, Chicago, IL, USA).

Data of in vitro experiments were expressed as mean ± SD or median ± IQR. After normalising variables, comparisons were conducted with a *t*-test or 2-way ANOVA using the GraphPad Prism 7.0 (GraphPad, San Diego, CA, USA) software.

## 3. Results

### 3.1. NR1D2 Regulates Growth and Apoptosis of CRC Cells

To study how NR1D2 affects CRC, we used an in vitro model with three commercially available isogenic cell lines: HCT-116 (p53 wild-type), HCT-116 p53 null (p53 silenced) and HCT-116 p53 mutated (knock-in of mutant at R248W p53 and knock-out of wild-type allele) in which this gene was silenced using siRNA technology.

**Figure 1:**
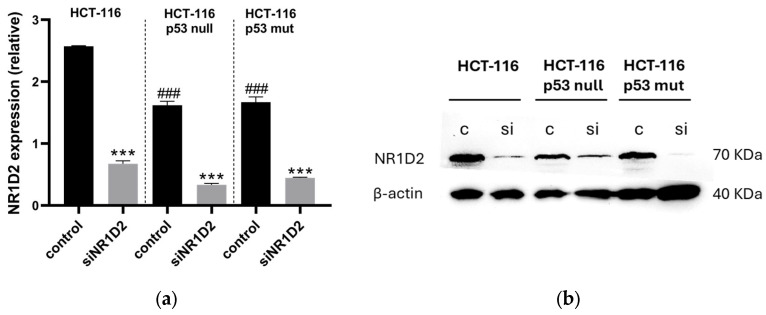
mRNA and protein expression of NR1D2 in the cell lines used in the in vitro model of this study, before and 72 h after transfection with siNRID2. Expression of the (**a**) mRNA and (**b**) protein in control and siNR1D2 transfected cells. *** *p* < 0.001 vs. control; ^###^ *p* < 0.001 vs HCT-116 cells.

As shown in Figure 1,tThe mRNA and protein expression of NR1D2 was similar in HCT-116 p53 null and HCT-116 p53 mutated control cells, and in both cases less than in HCT-116 (*p* < 0.001).

**Figure 2:**
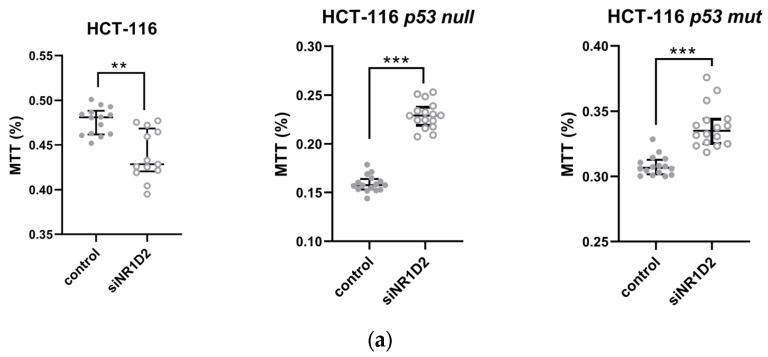

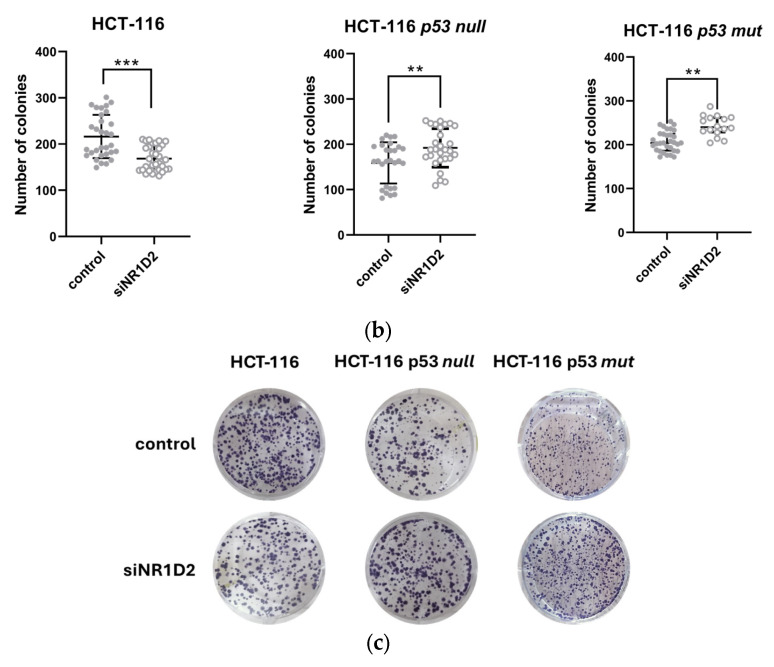
Regulation of cell growth on HCT-116, HCT-116 p53 null and HCT-116 p53 mut cells by NR1D2. (**a**) Cell viability obtained using the MTT assay, as described in the Materials and Method section. The results represented the median ± IQR of 3 experiments performed in quadruplicate. ** *p* < 0.01 vs. C; *** *p* < 0.001 vs. C. (**b**) Clonogenic assay was performed as described in Materials and Methods section to test the survival and proliferation capacity of cells. The results are presented as the median ± IQR of three experiments in triplicate. ** *p* < 0.01 vs. C; *** *p* < 0.001 vs. C. (**c**) A representative example of colony formation assay in control and siNR1D2 transfected HCT-116, HCT-116 p53 null and HCT-116 p53 mut cells.

Once the conditions for NR1D2 silencing were established, we analysed its effect on growth and viability of cells in vitro (Figure 2). The silencing of NR1D2 decreased the growth of HCT-116 cells and their capacity to form colonies, however it caused the opposite effect in the HCT-116 p53 null and HCT-116 p53 mutated cells (Figure 2a–c).

**Figure 3:**
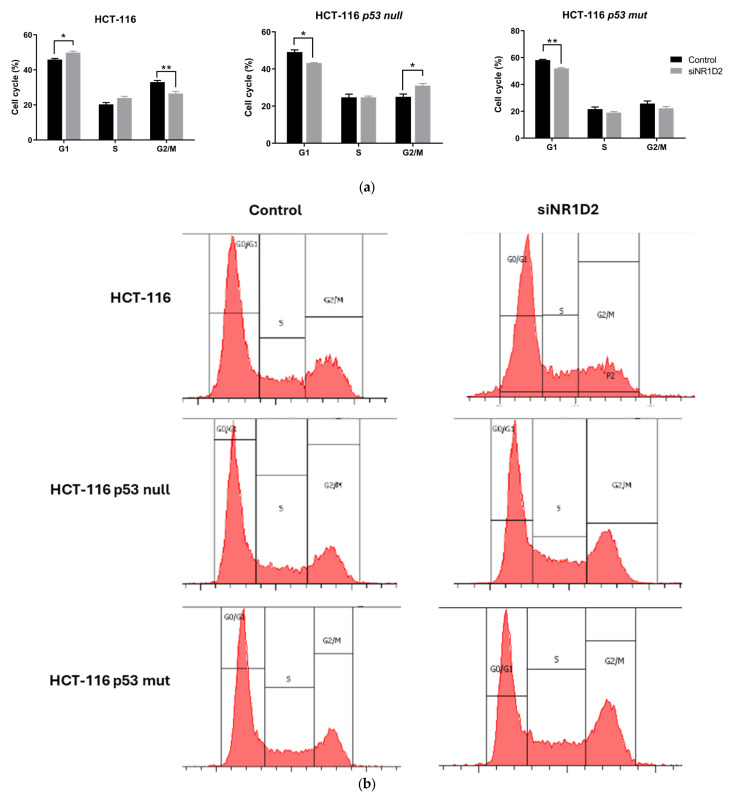
Regulation of cell cycle distribution by NR1D2 on HCT-116, HCT-116 p53 null and HCT-116 p53 mut cells. (**a**) Percentage distribution in different stages of cell cycle after NR1D2 silencing. Data represents the mean ± SD of three experiments performed in triplicate. ** *p* < 0.01 vs. control; * *p* < 0.05 vs. control. (**b**) Representative plots of control and transfected cells.

We next analysed the effects of NR1D2 on cell cycle distribution in the three different cell lines used in this study (Figure 3). The knock down of the NR1D2 gene induced accumulation in the G1 phase of the cycle (49.82 ± 2.52% of siNR1D2 transfected cells vs. 45.87 ± 1.62% of control cells) and a decrease in the percentage of cells in G2/M (26.50 ± 3.60% of siNR1D2 transfected cells vs. 33.05 ± 2.26% of control cells) in HCT-116 cells. Interestingly, silencing NR1D2 caused the opposite effect on HCT-116 p53 null cells, which accumulated in G2/M phase (31.00 ± 3.48% of siNR1D2 transfected cells vs. 25.03 ± 3.70% of control cells) and decreased in G1 (43.21 ± 0.46% of siNR1D2 transfected cells vs. 49.12 ± 2.96% of control cells). Similarly, transfection with siNR1D2 decreased in the percentage of cells in G1 (51.99 ± 0.93% of siNR1D2 transfected cells vs. 58.17 ± 0.87% of control cells), while no significant differences were observed in the other phases of the cycle.

**Figure 4:**
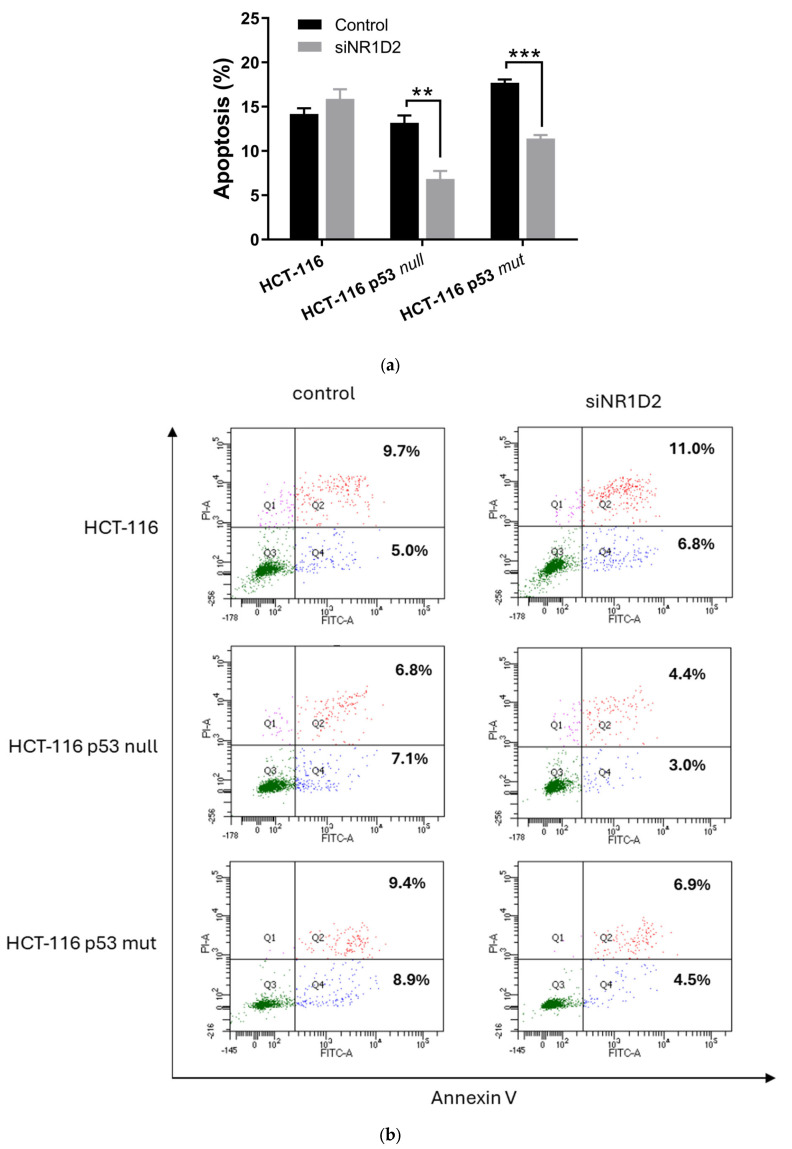
Effects of NR1D2 on cell death by apoptosis in CRC in vitro. (**a**) Levels of apoptosis of controls and transfected cells with siNR1D2 in HCT-116, HCT-116 p53 null and HCT-116 p53 mut cells. Results are presented as mean ± SD of three experiments performed in duplicate. ** *p* < 0.01 vs. control; *** *p* < 0.001 vs. control. (**b**) Representative plots of control a siNR1D2 transfected cells. Green: live cells; blue: apoptotic cells; red: late apoptotic cells.

We next studied whether NR1D2 regulates apoptotic death in HCT-116, HCT-116 p53 null and HCT-116 p53 mut (Figure 4). In HCT-116 p53 null and HCT-116 p53 mut cells, NR1D2 silencing induced a decrease in cell death by apoptosis, while no differences were found in HCT-116 cells.

### 3.2. Stemness Regulation by NR1D2 in CRC Cells In Vitro

**Figure 5:**
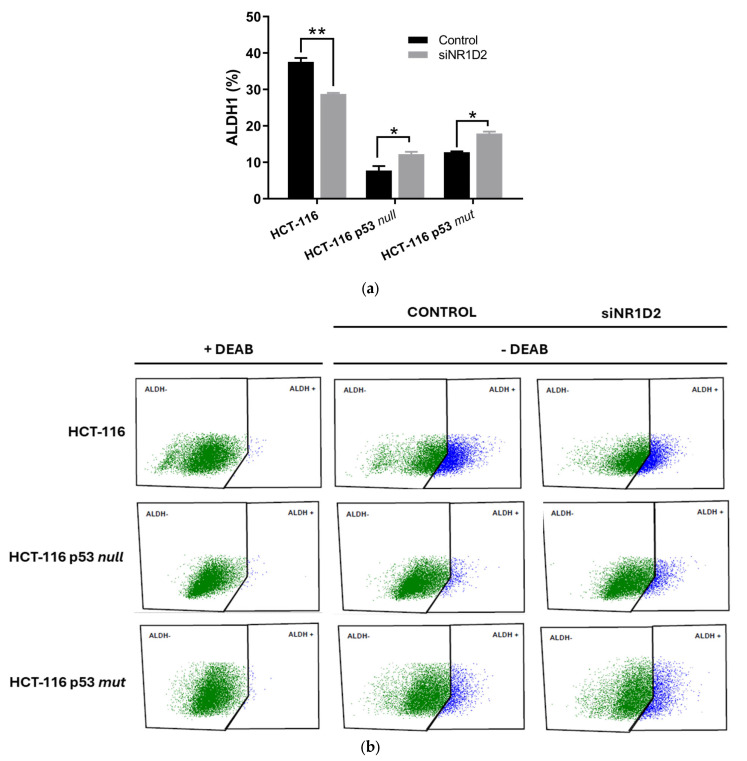
Influence of NR1D2 on ALDH1+subpopulation. (**a**) ALDH1+cells in HCT-116, HCT-116 p53 null and HCT-116 p53 mut cells before and after siNR1D2 transfection. Data represents the mean ± SD of three experiments performed in duplicate. * *p* < 0.05 vs. control; ** *p* < 0.01 vs. control. (**b**) Representative plots of different conditions studied.

To achieve whether the effects of NR1D2 on cell growth and apoptosis are related to the regulation of the amount and phenotype of CSCs in CRC, we determined ALDH1 + activity, the expression of CD326, CD44 and CD133 cell surface markers and the sphere formation capacity of cells, before and after NR1D2 knock-down. As shown in Figure 5a, transfecting cells with siNR1D2 decreased the ALDH1 + subpopulation in HCT-116 cells while increasing it in HCT-116 p53 null and HCT-116 p53 mut cells.

**Figure 6:**
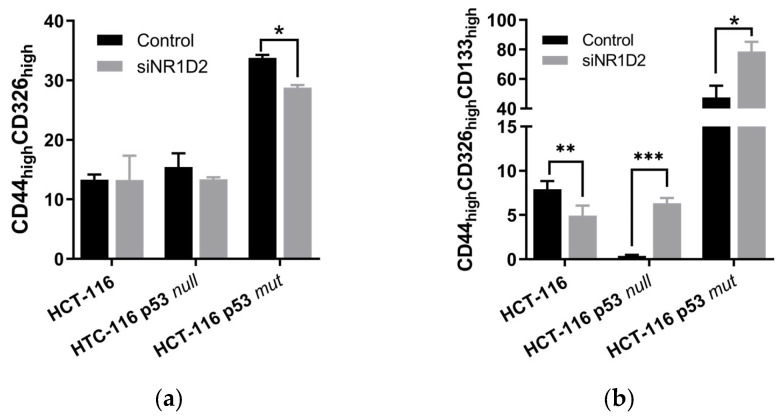
NR1D2 regulates the phenotypes of the CSC subpopulation in CRC. CD44_high_CD326_high_ (**a**) and CD44_high_CD326_high_CD133_high_ (**b**) subpopulations in HCT-116, HCT-116 p53 null and HCT-116 p53 mut cells after NR1D2 silencing. Data represents the mean ± SD of three experiments in duplicate. * *p* < 0.05 vs. control; ** *p* < 0.01 vs. control; *** *p* < 0.001 vs. control.

We next studied the phenotypes of CSCs after transfection with siNR1D2 through analysing the percentage of cells expressing CD44 and CD326 (CD44_high_CD326_high_) and CD44, CD326 and CD133 (CD44_high_CD326_high_CD133_high_). As shown in Figure 6, NR1D2 silencing in HCT-116 cells did not change the percentage of the CD44_high_CD326_high_ subpopulation, although it decreased the CD44_high_CD326_high_CD133_high_ subpopulation. In contrast, in the HCT-116 p53 null cell line, silencing NR1D2 increased in the CD44_high_CD326_high_CD133_high_ subpopulation, although no changes were seen in the CD44_high_CD326_high_ subpopulation. Finally, in HCT-116 p53 mut cells, NR1D2 knock down decreased the CD44_high_CD326_high_ subpopulation while increasing the CD44_high_CD326_high_CD133_high_ subpopulation.

**Figure 7:**
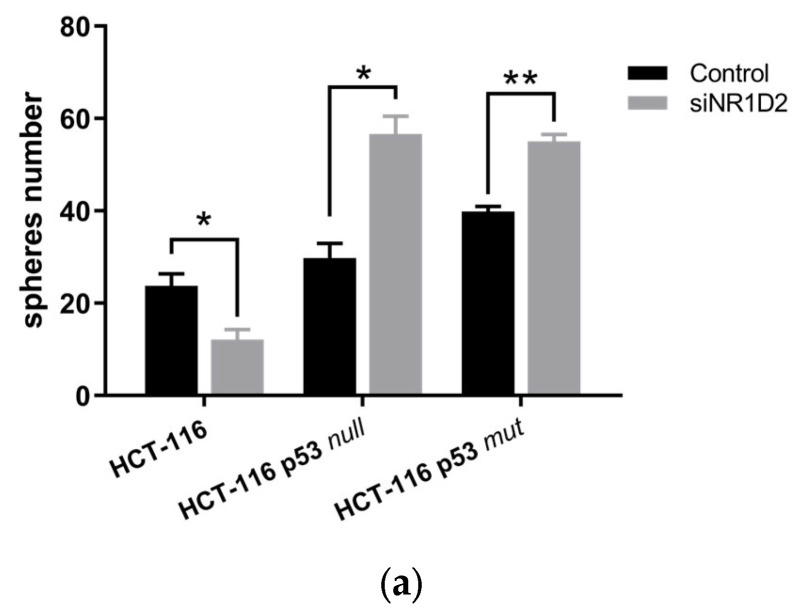

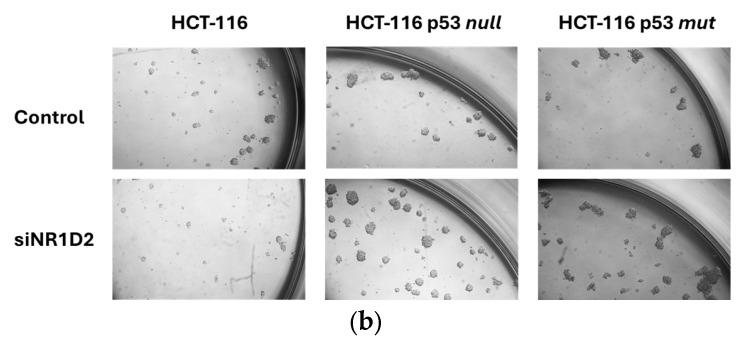
NR1D2 regulates the self-renewal capacity of the CSC subpopulation in CRC. (**a**) Spheroids obtained in HCT-116, HCT-116 p53 null and HCT-116 p53 mut cells after NR1D2 silencing. Data represents the mean ± SD of four experiments in quadruplicate. * *p* < 0.05 vs. control; ** *p* < 0.01 vs. control. (**b**) Representative images of spheroids formed from the three cell lines used in this study after NR1D2 silencing.

Finally, we studied the self-renewal capacity of cells after NR1D2 silencing analysing the sphere-forming faculty under free-serum conditions in an ultra-low anchorage-system. NR1D2 knock-down decreased the number of spheres formed by HCT-116 cells, while increasing it in HCT-116 p53 null and HCT-116 p53 mut cells (Figure 7).

### 3.3. NR1D2 Expression Correlates with Low Differentiation Grade in Tumour Tissues of CRC Patients

To further confirm the regulation of CSCs by NR1D2 in CRC, we measured NR1D2 mRNA expression in tissues from a cohort of 196 patients diagnosed with CRC (Table 1) in which we previously evaluated the status of p53 [19].

As shown in Table 2, NR1D2 expression increased in moderately and poorly differentiated versus well-differentiated tumours, although only in cases harbouring a wild type p53 (*p* = 0.043). No relationship was found between NR1D2 expression and other varibles studied (age, sex, tumour location (colon or rectum), T, N, M or pTNM stage).

**Figure 8:**
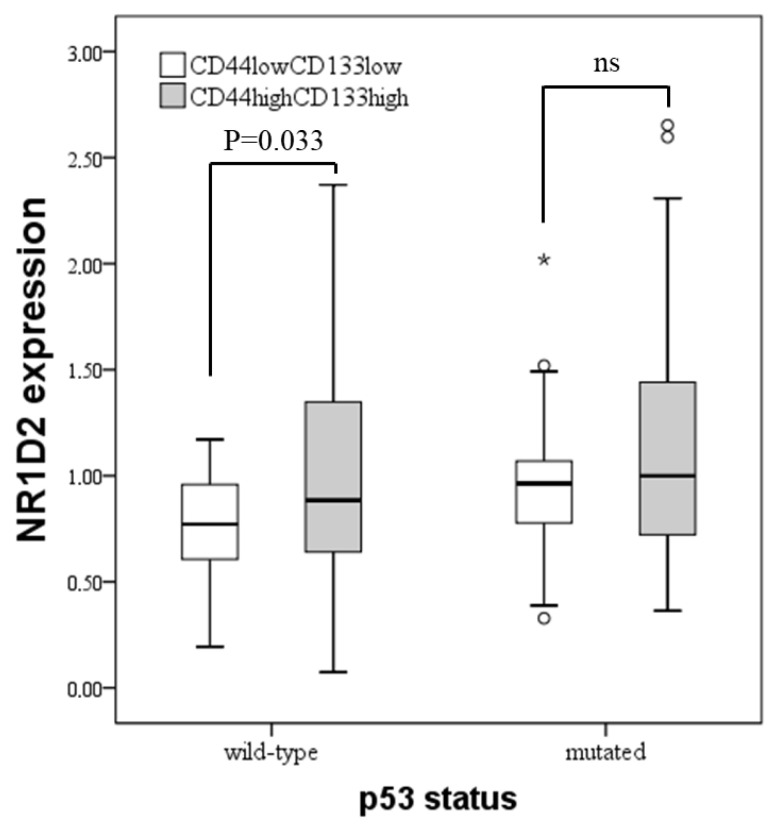
The relative mRNA expression of NR1D2 taking into account the status of p53 and the expression of CSCs markers in CRC samples from patients. Data represent the median ± IQR of data. * distant values more than 3 box lengths from 75th percentile; ° distant values more than 1.5 box lengths from 75th percentile. Ns: not significant

Finally, we studied the relationship between NR1D2 and CSCs in the CRC samples of the patients included in the study. In this cohort of patients, we had previously analysed the expression of CD44 and CD133 as markers of CSCs [19]. After stratifying patients into CD44_low_CD133_low_ and CD44_high_CD133_high_, we observed that NR1D2 expression increases in CD133_high_CD44_high_ tumours, although only in those harbouring wild type p53 (Figure 8).

## 4. Discussion

The physiology of the normal colon include several functions, such as colonic motor activity, colonic pressure, the absorption of nutrients or the expression of mucosal enzymes, that occurs in a circadian manner [22]. Even the composition of the gut microbiome seems to follows a circadian pattern [23]. Disruptions of these rhythms can cause several disorders, including cancer [24]. In fact, the involvement of the circadian clock in the development, progression and therapy resistance in cancer is widely accepted [25].

There are a wide range of studies in which most central circadian clock genes have been linked to different aspects of tumour biology. However, most of them focused on the Per1–3, and Cry1–2 genes or the transcription factors CLOCK and BMAL1. In contrast, very few reports have analysed the role of NR1D1–2 in the different hallmarks of cancer [11].

Recent results from our research group reported that NR1D2 can be considered as an independent prognostic factor for locoregional recurrence (LR) within 3 and 5 years after CRC diagnosis [14]. LR accounted for 4–11.5% of patients after curative resection for CRC, while distant metastasis appears in approximately 50% of CRC patients [26,27]. Both processes are attributed to CSCs [5]. According to this, NR1D2 silencing in cells harbouring wild type p53 decreased the ALDH1+cells and led to less aggressive phenotypes of CSCs. However, the opposite effect was found in cells without an active p53 and in cells harbouring a mutated one. As previously described, clock dysfunction can be pro- or anti-tumourigenic in a model- and cell-type specific manner [28]. Thus, the contradictory data found in our study could be related to the p53 status of the cell lines included in this study, due to the complex relationship between p53 and the molecular circadian clock. In fact, the p53 axis has been catalogued as the main link between the circadian clock and cancer biology [28]. The proteins of the circadian clock BMAL1, Per1, Per2, and Cry2 can interact with p53, regulating cell cycle, apoptosis and chromatin remodelling [29,30,31,32]. On the other hand, p53 can also block the binding of the CLOCK:BMAL1 dimer to the promoter of the Per2 gen, reducing its transcription [33]. According to our results, this relationship could extend to NR1D2, although more research is needed to uncover the mechanism involved.

CSCs are responsible for initiating and maintaining tumours. In addition, extrinsic and intrinsic apoptotic pathways are dysregulated in CSCs, making them more resistant to therapy [5]. In agreement with this, in our in vitro model, NR1D2 silencing in wild type p53 cells decreases cell growth, probably due to induced of cell cycle arrest with the accumulation of cells in the G0/G1 phase and increased apoptosis. In contrast, in cells lacking an active p53 or harbouring a mutated one, NR1D2 silencing increases proliferation, and reduces apoptosis. In glioblastoma CSCs, NR1D2 regulates proliferation through the AXL/PI3/Akt signalling pathways [34]. In this report, the authors also showed the activation of the AXL gene by NR1D2 in HCT-116 cells. It remains to be seen whether this is also the mechanism through which NR1D2 regulates growth, and apoptosis in p53 null and p53 mutated CRC cells. Other studies have revealed that NR1D2 plays a role in regulating the Hippo and Notch pathways which seems to be crucial for GSC-induced tumour growth [15].

Poorly differentiated CRC tend to grow and spread more quickly than well- and moderately differentiated ones, what has been associated with the presence of CSCs [35]. In our cohort, the high expression of NR1D2 correlated with undifferentiated tumours and high expression of CSCs markers, although only in tumours with a wild type p53. These results are consistent with those obtained using the in vitro model used in this study, which indicates that NR1D2 could be used as a target for treating this type of cancer in patients with tumours harbouring a wild type p53, and, therefore, to develop personalised medicine in CRC.

Synthetic NR1D1–2 ligands that pharmacologically target both receptors have resulted beneficial for treating sleep disorders as well as metabolic diseases [36]. Treating cancer cells with these compounds reduce the effectivity of chemotherapy with cisplatin [37] and increases cancer growth and metastasis [38] by regulating the lipid metabolism. Interestingly, these studies used in vitro models in which cells lines harbour mutated p53, supporting the results of our study, in which the silencing of NR1D2 resulted in greater growth in cells with a similar genic background. On the contrary, other studies suggest that NR1D1–2 agonists SR9009 and SR9011 acts as anticancer agents in both HCT-116 and HCT-116 p53 null cells, by regulating metabolism [39]. These contradictory results could be attributed to the NR1D1–2-independent effects on cell proliferation and metabolism of these compounds [40]. In addition, they act on both NR1D1 and NR1D2 [38,39,40], and, although redundant effects of both nuclear receptors on circadian rhythms regulation have been described [41], other studies have suggested differential roles in regulating physiological functions associated with the circadian clock [42].

NR1D2 could regulate stemness in cancer, probably through the regulation of glucose and lipid metabolism [15,43]. In fact, CSCs have several metabolic differences with the rest of the non-CSCs in the tumour. For example, they depend heavily on lipid metabolism to maintain their stem phenotype, with increased fatty acid oxidation, de novo lipogenesis, and cholesterol biosynthesis [44]. Glucose metabolism is also very important for CSCs, as they constantly alternate between glycolysis and oxidative phosphorylation to adapt to nutrient shortages and therapeutic stress, which is considered crucial to maintain their ability to self-renew [45]. NR1D2 has also been implicated in autophagy regulation [39], a highly conserved cellular process with a dual role in cancer, as it is involved in preventing tumour development in the early stages, as opposed to maintenance and metabolic adaptation in established tumours and metastasis [46]. The activation of autophagy may involve the degradation of a portion of cytosol, damaged organelles and proteins, misfolded proteins, or pathogens [46]. Autophagy regulates lipid [47] and glucose [48] metabolism in cancer cells. Therefore, this could be a mechanism of metabolic regulation by NR1D2 in cancer. Importantly, autophagy also regulates the state of quiescence in CSCs [30], a mechanism implicated in relapse in cancer [5,49] and highly dependent on autophagy [50]. Overall, NR1D2 could regulate stemness through autophagy and metabolism in CRC, although more research is guaranteed to demonstrate this mechanism.

The tumour microenvironment (TME) is thought to be essential in establishing and regulating CSCs [5]. The TME protects CSCs from immune surveillance, enhancing their invasive capacity and even promoting the dedifferentiation of non-stem cell-like cancer cells to refill the CSC pool. This interaction is reciprocal as CSCs can modify the phenotype of cells of the microenvironment [5]. Because of this, tumours can be considered as integrated ecosystems in which CSCs co-evolve with the TME [51]. In this sense, it has been reported that, circadian clocks of non-CSCs and stromal cells can regulate the circadian properties of CSCs [17]. Therefore, to analyse the influence of the circadian gene NR1D2 in tumour progression in depth, it is necessary to build more complex research models than those used in this study, such as 3D models, organoids from patients or transgenic animals.

In recent years, circulating tumour DNA (ctDNA) has become an excellent tool for CRC diagnosis and treatment optimisation [52], as it can present relevant information such as tumour burden [53], tumour-specific genomic alterations [54], and tumour heterogeneity [55]. Indeed, it has been reported that it could be particularly useful for decision-making in the adjuvant treatment of this type of tumour in stages II/III [56], which remains quite controversial [57]. Most studies have focused on determining the amount of ctDNA present (ctDNA positive vs. ctDNA negative) at certain points after treatment, which has also helped the prognosis of relapse [56]. These results, together with the possibility of determining p53 mutations and genic variants (including those of NR1D2) [52], as well as CSC-ctDNA [58], could mean a step forward in the diagnosis of tumour progression as well as response to treatment in CRC.

## 5. Conclusions

NR1D2 could be considered a pharmacological target for the treatment of CRC, although only in the cases of patients with tumours harbouring a wild type p53, since its silencing decreases growth and increases cell death by apoptosis. This appears to be because NR1D2 regulates the amount and phenotype of CSCs. However, more studies are needed to determine the mechanism by which NR1D2 regulates stem character in this type of cancer. However, it is necessary to gain an in-depth understanding of the mechanism by which NR1D2 regulates growth and progression in CRC. For this reason, studies with more complex models are required, such as three-dimensional cultures, transgenic mice or organoids from patients, including stromal cells, such as fibroblasts or immune cells, as their influence on the circadian characteristics of CSCs has been demonstrated [17].

## Figures and Tables

**Table 1:** The characteristics of the patients included in this study.

Characteristics		N (%)
**Age**	<72	92 (46.9)
	≥72	104 (53.1)
**Sex**	Female	74 (38.1)
	Male	122 (61.9)
**Location**	Colon	184 (93.4)
	Rectum	12 (6.6)
**DG ^1^**	Well differentiated	43 (21.9)
	Moderately differentiated	131 (66.8)
	Poorly differentiated	22 (11.3)
**T stage**	T1	4 (2.0)
	T2	26 (13.27)
	T3	126 (64.3)
	T4	40 (20.4)
**N stage**	N0	103 (52.6)
	N1	50 (25.5)
	N2	43 (21.9)
**M stage**	M0	177 (90.3)
	M1	19 (9.7)
**pTMN stage**	Stage I	22 (11.3)
	Stage II	74 (37.9)
	Stage III	82 (41.5)
	Stage IV	18 (9.2)

^1^ Diferentiation grade.

**Table 2:** The relationship between NR1D2 expression and the characteristics of the CRC patients included in this study.

		All ^1^		wtp53 ^2^		mtp53 ^3^	
Characteristic		Median ± IQR ^5^	*p*	Median ± IQR ^5^	*p*	Median ± IQR ^5^	*p*
**Age** (**y**) *^4^	<72	0.90 (0.62–1.27)	0.453	0.83 (0.68–1.11)	0.509	0.93 (0.79–1.41)	0.704
	≥72	0.85 (0.54– 1.21)		0.84 (0.51–1.17)		0.99 (0.65–1.27)	
Sex *	Male	0.92 (0.58–1.28)	0.077	0.90 (0.52–1.24)	0.442	1.01 (0.79–1.41)	0.077
	Female	0.75 (0.56–1.07)		0.74 (0.66–1.04)		0.90 (0.48–1.18)	
Location *	Colon	0. 89 (0.58–1.22)	0.508	0.84 (0.58–1.16)	0.353	0.95 (0.65–1.31)	0.352
	Rectum	0.86 (0.42–1.52)		0.62 (0.42–1.30)		1.50 (0.86–1.27)	
**DG ^†&^**	Well	0.72 (0.44–1.17)	0.380	0.61 (0.43–0.85)	**0.043**	0.95 (0.46–1.58)	0.780
	Moderately	0.91 (0.59–1.23)		0.86 (0.62–1.19)		1.01 (0.74–1.40)	
	Poor	0.90 (0.69–1.25)		0.97 (0.68–1.28)		0.91 (0.89–1.40)	
T stage *	T1+T2	1.02 (0.73–1.34)	0.191	0.80 (0.64–1.08)	0.772	1.18 (0.97–1.74)	0.257
	T3+T4	0.87 (0.55–1.19)		0.83 (0.53–1.17)		0.92 (0.58–1.30)	
**N stage ^†^**	N0	0.81 (0.53–1.14)	0.361	0.74 (0.49–1.15)	0.875	0.92 (0.54–1.17)	0.075
	N1	0.89 (0.52–1.29)		0.70 (0.52–1.29)		0.90 (0.67–1.77)	
	N2	0.97 (0.78–1.23)		0.92 (0.73–1.00)		1.16 (0.92–1.56)	
M stage *	M0	0.89 (0.57–1.23)	0.927	0.84 (0.53–1.18)	0.685	0.96 (0.76–1.40)	0.719
	M1	0.85 (0.63–1.20)		0.81 (0.61–1.01)		1.06 (0.56–1.56)	
pTNM Stage *	Stage I+II	0.94 (0.71–1.14)	0.080	0.79 (0.52–0.99)	0.427	1.02 (0.91–1.32)	0.097
	Stage III+IV	0.91 (0.64–1.27)		0.88 (0.63–1.17)		1.11 (0.72–1.35)	

* Analysis was performed using the non-parametric Mann–Whitney U test for independent samples or the **^†^** Kruskal–Wallis test for independent samples; ^&^ Differentiation Grade; ^1^ All cases studied; ^2^ p53 wild-type tumours; ^3^ p53 mutated tumours; ^4^ Stratified by the median; ^5^ Interquartile range.

## Data Availability

The original contributions presented in this study are included in the article/Supplementary Materials. Further inquiries can be directed to the corresponding author.

## Supplementary Materials

The following supporting information can be downloaded at: https://www.mdpi.com/article/10.3390/biomedicines13061500/s1, Table S1: Primers used to analyse P53 mutations; Table S2: TP53 mutations observed in cancer patients. Table S3: Primers used to determine NR1D2, CD44, CD133, UBC, TBP and RPS13 expression.

## Abbreviations

The following abbreviations are used in this manuscript:

CSCs	Cancer stem cells
CICs	Cancer-initiating cells
CRC	Colorectal cancer
NR1D2	Nuclear Receptor Subfamily 1 Group D Member 2
SCN	Suprachiasmatic nucleus
RHT	Retino-hypothalamic tract
TTFL	Transcription/translation feedback loops
CCG	Clock-controlled genes
TME	Tumour microenvironment
UBC	Ubiquitin C
TBP	TATA binding protein
RSP13	Ribosomal protein S13
PI	Propidium iodide
ALDH1	Aldehyde dehydrogenase 1
PE	Phycoerythrin
APC	Allophycocyanin
FITC	Fluorescein IsoTioCyanate
