# Supplementary material for: Regulation of Stemness by NR1D2 in Colorectal Cancer
Source: Biomedicines. 2025 Jun 18;13(6):1500. doi: 10.3390/biomedicines13061500 (PMC12191377; doi:10.3390/biomedicines13061500)
Supplement: Supplementary file 1 [file biomedicines-13-01500-s001.zip › biomedicines-3656851-supplementary.pdf]

Supplementary table 1. Primers used to analyze P53 mutations

TP53	Forward	Reverse
Exon 2-4	agctgtctcagacactggcatggtgttgg	cactgacaggaagccaaagggtgaagagg
Exon 5-6	gttgctttatctgttcacttgtgccctgac	tagggaggtcaatatagcagcaggagaaag
Exon 7-9	cagcctgggcgacagagcgagattccatc	aaccaggagccattgtctttgaggcatcac
Exon 10	tacttgaagtgcagtttctactaaatgcatg	aggaagactaaaaaaatgtctgtgcagggc

Supplementary table 2. TP53 mutations observed in cancer patients

Type of TP53 mutation[a]		Exon	No. of patients
c.375G>A (p.T125=)[b]	Synonymous	4	1
c.391A>T (p.N131Y)	Missense	5	1
c.404G>T (p.C135F)	Missense	5	2
c.423C>G (p.C141W)	Missense	5	1
c.438G>A (p.W146X)	Nonsense	5	2
c.455_455delC (p.(P152Rfs*18)	Frameshift	5	1
c.455C>T (p.P152L)	Missense	5	2
c.481G>T (p.A161S)	Missense	5	1
c.510G>A (p.T170=)[c]	Synonymous	5	1
c.517G>T (p.V173L)	Missense	5	1
c.515T>G (p.V172G)	Missense	5	1
c.520A>T (p.R174W)	Missense	5	1
c.524G>A (p.R175H)	Missense	5	8
c.527G>A (p.C176Y)	Missense	5	1
c.527G>T (p.C176F)	Missense	5	1
c.536A>G (p.H179R)	Missense	5	1
c.569C>T (p.P190L)	Missense	6	1
c.584T>C (p.I195T)	Missense	6	1
c.592G>T (p.E198X)	Nonsense	6	1
c.632C>T (p.T211I)	Missense	6	1
c.635_636delTT (p.(F212Sfs*3))	Frameshift	6	1
c.637C>T (p.R213X)	Missense	6	5
c.652_654delGTG (p.V218del)	Frameshift	6	1
c.659A>G (p.Y220C)	Missense	6	1
c.672+2T>C (p ?)		Intron 6	1
c.711G>A (p.M237I)	Missense	7	1
c.713G>A (p.C238Y)	Missense	7	2
c.720_721delTTinsCC (p.(S241P))	Frameshit	7	1
c.733G>A (p.G245S)	Missense	7	4
c.734G>A (p.G245D)	Missense	7	2
c.742C>T (p.R248W)	Missense	7	4
c.743G>A (p.R248Q)	Missense	7	4
c.745A>T p.R249W	Missense	7	1
c.775G>T (p.D259Y)	Missense	7	1
c.782G>A (p.S261N)	Missense	8	1
c.783-1G>T (p?)		Intron 7	1
c.811G>A (p.E271K)	Missense	8	1
c.814G>A (p.V272M)	Missense	8	2
c.817C>T (p.R273C)	Missense	8	4
c.818G>A (p.R273H)	Missense	8	6
c.818G>C (p.R273P)	Missense	8	1
c.819_826dupTGTTTGTG	Frameshift	8	1
c.820G>T (p.V274F)	Missense	8	1
c.844C>T (p.R282W)	Missense	8	1
c.853G>A (p.E285K)	Missense	8	1
c.904G>T (p.G302W)[d]	Missense	8	1
c.916C>T (p.R306X)	Nonsense	8	1

[a] Mutations are reported according to Recommendations of the Human Genome Variation Society (www.hgvs.org). [b] This synonymous mutation was considered and calculated as mutated because it is known to impair TP53 splicing (0 predicted activity of protein). [c] Mutation was considered and calculated as wild-type as it was characterized as silent mutation without affecting transcription, splicing or translation. [d] Mutation was considered and calculated as wild-type due to transactivation activity > 75%.

Supplementary table 3. Primers used to determine NR1D2, CD44, CD133, UBC, TBP and RPS13 expression

Gene	Forward	Reverse
NR1D2	acttgaagctattctggaactaacat	catgtcctcatcaattacagtttgtag
CD44	gctttcaatagcaccttgcccacaatgg	aaagaggtcctgtcctgtccaaatcttcc
CD133	tccacagaaatttacctacattgg	cagcagagagcagatgacca
UBC	tgggatgcaaatcttcgtgaagaccctgac	accaagtgcagagtggactctttctggatg
TBP	ttgtcctttttgccatttgctgggctcctc	gtcatcagtggagacggtttccatttaacc
RPS13	ggtgttgcacaagtacgttttgtgacaggc	tcatatttccaattgggagggaggactcgc
